# The Longitudinal Association Between Internet Addiction and Prosocial Behavior Among Chinese Adolescents: Testing a Moderated Mediation Model

**DOI:** 10.3390/bs15030322

**Published:** 2025-03-06

**Authors:** Wei-Xuan Liang, Wan-Yu Ye, Kai-Xin Ng, Kai Dou, Zhi-Jun Ning

**Affiliations:** 1 Department of Psychology and Research Center of Adolescent Psychology and Behavior, School of Education, Guangzhou University, Guangzhou 510006, China; 2112208036@e.gzhu.edu.cn (W.-X.L.); 2112108042@e.gzhu.edu.cn (W.-Y.Y.); 2112308204@e.gzhu.edu.cn (K.-X.N.); 2 Mental Health and Artificially Intelligent Intervention Research Center of Guangzhou, Guangzhou University, Guangzhou 510006, China

**Keywords:** internet addiction, prosocial behavior, self-control, peer rejection, adolescents

## Abstract

Internet addiction has been associated with decreased prosocial behavior in adolescents, and minority studies have investigated the underlying mechanisms involved. This study aimed to examine the mediating effects of self-control and the moderating effects of peer rejection. A longitudinal study with two waves (6 months apart) was used to measure internet addiction (T1), peer rejection (T1), self-control (T1/T2), and prosocial behavior (T1/T2) among 1048 secondary school students (*M*_age_ = 14.80 years old, *SD* = 1.61) in a southern Chinese metropolitan area. A longitudinal path analysis model was applied to analyze the data and derive insights about the relationships between these variables. The findings indicated that T1 internet addiction negatively influenced later prosocial behavior through reduced self-control, particularly among adolescents with lower levels of peer rejection. These findings clarify how internet addiction impairs prosocial development, and we propose a framework for intervention: mitigating peer rejection and harnessing self-control as a mediator to counteract the adverse effects of internet addiction.

## 1. Introduction

Prosocial behavior plays a vital role in adolescents’ social development. It refers to actions in which individuals willingly offer assistance or confer benefits to others (Eisenberg & Mussen, 1989; Yin et al., 2022), encompassing behaviors such as cooperation, sharing, helping, and caring (Dou et al., 2019). In recent years, increasing reliance on digital technologies, particularly smart devices and the internet, has raised concerns about its potential negative impact on adolescents’ prosocial development (Benedetto et al., 2024; Collins & Freeman, 2013; Z. Wang et al., 2020; Yang et al., 2017). The issue of internet addiction has become a significant societal concern, particularly in the context of Chinese adolescents, where internet use has become an integral part of daily life (Yang et al., 2017). Studies indicate that internet addiction is significantly correlated with negative social and psychological outcomes, such as lower self-esteem and social isolation (C. Li et al., 2014). Despite the increasing attention to internet addiction, the bulk of existing research has primarily focused on the negative consequences of internet addiction, such as aggressive behavior and social withdrawal, while the effects on adolescents’ prosocial behaviors remain under-explored (Chou et al., 2017; Dou et al., 2020; Zhai et al., 2019).

The mechanism underlying the relationship between internet addiction and prosocial behavior remains complex. A key factor that may mediate this relationship is self-control, which has been shown to be significantly associated with both internet addiction and prosocial behavior (C. Li et al., 2014). Adolescents with lower levels of self-control are more likely to engage in excessive internet use, which in turn may diminish their ability to regulate their behaviors and engage in prosocial actions offline (Rankin, 2019). Furthermore, the peer environment plays a crucial role in shaping adolescents’ behavior (Chávez et al., 2022; Loparo et al., 2023; Xiao & Qian, 2023). Negative peer relationships, such as peer rejection, have been linked to both self-control difficulties and an increase in externalizing behaviors, including aggression and social withdrawal (Meldrum & Hay, 2012; Loparo et al., 2023). However, the moderating role of peer rejection in the relationship between internet addiction and prosocial behavior remains under-explored. To address these gaps, two-time longitudinal data were used to examine the association between internet addiction and adolescent prosocial behavior, and to examine self-control as a mediator and peer rejection as a moderator.

### 1.1. Internet Addiction and Prosocial Behavior

Internet addiction is defined as the compulsive and excessive use of the internet, leading to a loss of control over online activities (Zhou et al., 2022). Adolescents perceive activities such as chatting and online games as essential parts of their daily life, and their engagement in using the internet has grown substantially over the past decade (Subramaniam, 2014). Evidence indicates that internet addiction is associated with a range of negative psychological, social, and physical consequences, including impacts on adolescents’ mental health and social relationships (Van Der Graaff et al., 2018; Zhao & Pan, 2022; Zhou et al., 2022). While most research on internet addiction has focused on its negative consequences, there is a growing recognition of its potential to inhibit positive behaviors, particularly prosocial actions.

Adolescents’ prosocial behaviors are crucial to their psychological well-being, contributing to higher self-esteem, life satisfaction, and overall mental health (Dou et al., 2019; Yang et al., 2017; Zuffianò et al., 2016). Based on self-determination theory (Ryan & Deci, 2000), intrinsic motivation is identified as the fundamental form of motivation behind internet games. While internet addiction satisfies adolescents’ intrinsic needs for pleasure and satisfaction, it accomplishes this by emphasizing individual rewards from the virtual world (Ryan et al., 2006). This over-reliance on online activities reduces adolescents’ sense of autonomy and limits opportunities for real-world social interactions (Meshi & Ellithorpe, 2021). As a result, despite fulfilling intrinsic motivations, internet addiction can reduce prosocial motivation and behaviors in offline life.

Among the limited number of studies on this topic, Collins and Freeman (2013) reported a negative relationship between the problematic use of video games and prosocial tendencies. Yang et al. (2017) reported that internet addiction in Chinese adolescents affects adolescents’ long-term psychological functions and well-being. Z. Wang et al. (2020) found that prosocial peers can provide emotional support for adolescents, thereby mitigating adolescents’ cybervictimization. García-Gil et al. (2022) further reported a negative association between adolescents’ problematic video game use and prosocial behaviors. Benedetto et al. (2024) explored the relationship between internet addiction and prosocial behavior using a cross-sectional study. However, these studies lacked a unified terminology for internet addiction, and they were primarily cross-sectional. Therefore, longitudinal research is essential for examining the correlation between internet addiction and prosocial behavior.

### 1.2. The Mediating Effect of Self-Control

Self-control is the ability to control or regulate individuals’ emotions, cognition, and behaviors (Kim et al., 2017), and is generally associated with social norms and achievement goals (Dou et al., 2019). According to self-control theory (Gottfredson & Hirschi, 1990), individuals tend to impair their self-control resources during decision-making processes (Alós-Ferrer et al., 2015). Consistently, Telzer et al. (2011) reported that individuals often rely on their self-control abilities to resist immediate desires and to make prosocial decisions. Joosten et al. (2015) also indicated that a depletion of self-control decreases engagement in prosocial behavior. Therefore, we argue that individuals with low levels of self-control exhibit less prosocial behavior.

Additionally, internet addiction can also be seen as leading to a loss of individual self-control ability on the internet. Indulging in the internet world will lead to weakened self-control and a state of self-exhaustion (J. Li et al., 2021). Specifically, internet addictions exhaust more cognitive resources to effectively complete impulse control tasks, which can impair the function of relevant areas of the brain, leading to an impairment of decision making and control (C. Li et al., 2014). Furthermore, prior research has demonstrated a detrimental association between self-control and internet addiction (Kim et al., 2017; C. Li et al., 2014; Özdemir et al., 2014).

Overall, we hypothesize that internet addiction is associated with less prosocial behavior in adolescents via low levels of self-control. Self-control may mediate the relationship between internet addiction and prosocial behavior, but this notion has been relatively overlooked and requires further investigation.

### 1.3. The Moderating Effect of Peer Rejection

Individuals generally spend greater amounts of time with their peers than in other social environments (i.e., family) during adolescence (King et al., 2018). This period is marked by increasing independence. Adolescents are inclined to establish their presence within their peer groups in order to obtain stable and long-term norms and support (Sussman et al., 2007). Peer systems can influence adolescents both directly and indirectly. Positive peer relationships play a vital role in fostering adolescent development through academic support, a better perception of subjective well-being, and prosocial behavior (Alsarrani et al., 2022; Lessard & Juvonen, 2022; Zhou et al., 2022). However, negative peer relations such as peer rejection can lead to serious consequences, such as internalizing and externalizing problems (Loparo et al., 2023; Xiao & Qian, 2023).

Peer rejection is the persistent social experience of adolescents experiencing chronic exclusion from their peer group (Xiao & Qian, 2023), encompassing neglect and victimization (Loparo et al., 2023). Meldrum and Hay (2012) underscore the pivotal role of peer relationships in shaping self-control. Adolescents’ ability to self-regulate is diminished when they experience rejection from their peers. Deficiencies in self-control ability probably result in negative outcomes, such as aggressive behavior, mental problems, and acting recklessly (Meldrum & Hay, 2012; Zhao & Jin, 2023). In addition, Huang et al. (2023) reported that adolescents with poor interpersonal relationships tend to dedicate more time to internet usage and exhibit greater tendencies towards addictive behaviors. When adolescents experience rejection from their peers, they tend to become addicted to the internet as a way to compensate for the absence of realistic social relationships (P. Wang et al., 2017; Zhao & Jin, 2023).

Similarly, Yu et al. (2021) discovered that peer influences and self-control serve as risk factors for internet gaming disorder. Their joint effect exacerbates the maladaptive cognitions related to internet gaming, consequently increasing the risk of internet addiction. However, studies on the moderating effect of peer rejection on internet addiction and self-control are limited. There is also a lack of longitudinal studies that construct comprehensive explanatory models to understand how the dual cumulative risk factors for peer rejection and internet addiction affect self-control, thereby weakening prosocial behavior.

### 1.4. The Present Study

**Figure 1:**
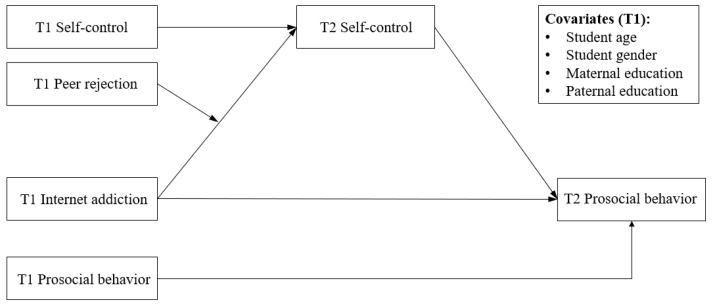
Conceptual moderated mediation model. Note: T1 = Time 1; T2 = Time 2. Same as below.

Although the negative effect of internet addiction on adolescents’ prosocial behaviors is widely acknowledged, few studies have investigated the specific effects of internet addiction on adolescents’ prosocial behaviors through the lens of self-control ability. On the basis of the research reviewed, this study tested a two-time longitudinal design to construct a moderated mediation model, as depicted in Figure 1. We formulated the following hypotheses: internet addiction is negatively associated with adolescents’ prosocial behaviors (Hypothesis 1); self-control mediates the association between internet addiction and prosocial behavior (Hypothesis 2); (3) peer rejection moderates the association between internet addiction and self-control (Hypothesis 3a); and peer rejection moderates the mediating effect of self-control (Hypothesis 3b).

## 2. Methods

### 2.1. Participants and Procedures

The participants were 1048 adolescents (571 males and 477 females) from three high schools (*M*_age_ = 14.80 years old, *SD* = 1.61) in Guangzhou, who were enrolled via a convenience sampling approach. We collected two waves of data in June 2019 (Time 1) and December 2019 (Time 2). At Time 1, the adolescents were between 11 and 19 years of age (*M*_age_ = 14.80, *SD* = 1.61). The majority of the participants’ fathers (90.6%) and mothers (80.4%) held a middle school diploma at least. At Time 2, 951 adolescents (524 males and 427 females) participated in the second wave (attrition rate = 9.30%). Attrition occurred because some participants were absent on the day of data collection.

The Guangzhou University Research Ethics Committee approved all procedures involving human participants (Protocol Number: GZHU2019018). In the written informed consent forms, which told them that their participation was voluntary and that they could withdraw from the survey at any time, all of the adolescents and their parents signed their consent to participate in the study. All the questionnaires were administered in the classroom using paper and a pencil. The surveys were facilitated by two trained research assistants who were able to provide clarification to participants if needed. The data were collected during regular class hours. Please see all the measurement scales in Appendix A.

### 2.2. Measures

#### 2.2.1. Internet Addiction

We defined negative internet use as internet addiction in this study, but the terminology in the academic literature is inconsistent due to the diversity of the field. Thus, we measured internet addiction at T1 with the Young Diagnostic Questionnaire (Young, 2004). The scale has 8 items measuring adolescents’ symptoms of internet addiction (e.g., “Do you stay online longer than originally intended?”) that are binarily rated (0 = *No*/1 = *Yes*). The YDQ scores were obtained using the division method, which involves the division of the total scores by the number of items. Higher scores indicate more internet addiction symptoms. Cronbach’s alpha for this scale was 0.78 at T1.

#### 2.2.2. Peer Rejection

Adolescents’ peer rejection was measured by the peer rejection questionnaire at T1 using the social exclusion scale compiled by Thau et al. (2015). The title description was slightly modified to define peers as the perpetrators of exclusion and to ensure relevance to the adolescents. The scale has 6 items (e.g., “I sometimes feel as if some of my companions are ignoring me”) rated on a five-point scale (from “1 = *totally disagree*” to “5 = *totally agree*”). The total scores for the questionnaire were calculated by dividing the sum of the scores by the number of items, with higher scores indicating higher levels of perceived peer rejection by adolescents. Cronbach’s alpha for this scale was 0.91 at T1.

#### 2.2.3. Self-Control

Participants’ self-control at both T1 and T2 was assessed with the Brief Self-Control Scale (Tangney et al., 2004). This scale has been used within the Chinese cultural context and has been proven to be applicable to Chinese adolescents (Dou et al., 2020; Situ et al., 2016). This scale consists of 13 items (e.g., “I have a hard time breaking bad habit”) rated on a five-point scale (from “1 = *not like me at all*” to “5 = *very much like me*”). The BSCS scores were calculated by dividing the total scores by the number of items, with higher scores indicating a greater self-control ability. Cronbach’s alphas were 0.79 at T1 and 0.82 at T2 in the current study.

#### 2.2.4. Prosocial Behavior

Prosocial behavior at both T1 and T2 was measured with the 5-item prosocial behavior scale (Goodman et al., 1998). A sample item is as follows: “I try to be nice to other people, and I care about their feelings”. The items are rated on a three-point scale (1 = *not true*; 2 = *somewhat true*; 3 = *certainly true*). Greater prosocial behavior tendencies were indicated by higher scores, which were calculated by dividing the overall score by the total number of items in the questionnaire. For this study, Cronbach’s alpha was 0.76 at T1 and 0.85 at T2.

#### 2.2.5. Covariates at T1

All of the analyses included the following covariates: student age, student sex (1 = *male*; 2 = *female*), and mother’s and father’s educational background (1 = *primary school*; 2 = *middle school*; 3 = *undergraduate*; 4 = *graduate*).

### 2.3. Data Analysis Plan

The data were analyzed in several steps. First, we conducted attrition analysis, descriptive statistics, and bivariate correlations for the study variables with IBM SPSS 26.0. Following this, considering that self-report measures were being used, we utilized Harman’s single-factor test and confirmatory factor analysis (CFA) to check for common method bias. We subsequently used a longitudinal path analysis model (LPAM) to analyze the moderated mediation model with Mplus 8.3 (Muthén & Muthén, 2012). Adolescents’ internet addiction at T1 and prosocial behaviors at T2 were included in the mediation model as the independent and dependent variables, respectively, with self-control at T2 acting as the mediator for testing Hypotheses 1 and 2. Then, in order to test Hypothesis 3, we integrated the moderator (i.e., T1 peer rejection) into the aforementioned mediation model. Prior to producing the interaction terms, all variables were centered in the context of the moderated mediation model. We continued on to a simple slope analysis when the interaction effect became significant. This involved testing the mediating effect of T2 self-control when the levels of the moderator (i.e., T1 peer rejection) were one standard deviation below or above the mean. This allowed us to explore the extent to which peer rejection moderated the association between T2 self-control and T2 prosocial behavior. Furthermore, it enabled us to assess the moderation effects of peer rejection on the mediation of self-control.

To assess the model fit, we relied on the comparative fit index (CFI; acceptable > 0.90, good > 0.95), root mean square error of approximation (RMSEA; acceptable < 0.08, good < 0.05; Steiger, 1990), and standardized root mean square residual (SRMR; acceptable < 0.08, good < 0.05) values. We used a bootstrapping technique (*N* = 5000) and 95% confidence intervals (CIs) to determine the significance of the (moderated) mediation effect. A (moderated) mediation effect was considered significant when the 95% CI did not include 0.

## 3. Results

### 3.1. Attrition Analysis

Attrition analyses were conducted to examine potential bias between participants who completed measures across time points (Group 1) and participants who dropped out at T2 (Group 2). The results revealed no significant differences between the two groups in terms of sex (*χ*^2^(1) = 1.568, *p* = 0.211), father’s education (*χ*^2^(3) = 1.055, *p* = 0.788), mother’s education (*χ*^2^(3) = 6.660, *p* = 0.084), T1 internet addiction (*t* (1046) = 0.282, *p* = 0.778), T1 peer rejection (*t* (1046) = 0.978, *p* = 0.328), T1 self-control (*t* (1046) = 0.800, *p* = 0.424), or T1 prosocial behavior (*t* (1046) = 1.263, *p* = 0.207). However, a difference was observed between the two groups in terms of age (*t* (1028) = −2.426, *p* = 0.015). Thus, we created a variable indicating the presence or absence of missing data and examined its correlation with the main outcome variables. These correlations were found to be small and nonsignificant. Thus, the results suggested that the data could be considered missing at random (MAR). In summary, these results indicated that the data set was unlikely to be biased due to attrition.

### 3.2. Common Method Bias

First, common method bias is a potential issue for adolescents (Podsakoff et al., 2003). We utilized Harman’s single-factor test to check for common method bias (Harman, 1976). The results showed that the first factor explained only 18.48% of the variance, which is less than the 50% threshold. Second, confirmatory factor analysis (CFA) was employed to evaluate the validity of the hypothesized factor structure. Specifically, we compared a six-factor model, based on the six primary study variables, with a one-factor model that included all the self-assessment items to assess which model best fit the data and to provide empirical support for the proposed factor structure (Conway & Lance, 2010). The results indicated that the six-factor model yielded acceptable fit indices: normed Chi-square (*χ*^2^/df) = 2.844, TLI = 0.846, CFI = 0.854, RMSEA = 0.042. Furthermore, the one-factor model exhibited poor fit indices: *χ*^2^/df = 8.428, TLI = 0.378, CFI = 0.403, RMSEA = 0.084. As a result, common method bias did not pose a substantial threat to the interpretation of the results.

### 3.3. Descriptive and Correlations

The descriptive statistics and correlations among the primary study variables are presented in Table 1. Specifically, T1 internet addiction was positively related to T1 peer rejection and negatively related to T2 self-control and T1/T2 prosocial behavior. In addition, T1 peer rejection was negatively related to T2 self-control and T1/T2 prosocial behavior. Moreover, T2 self-control was positively related to T1/T2 prosocial behavior.

### 3.4. Mediating Effects of Self-Control

**Figure 2:**
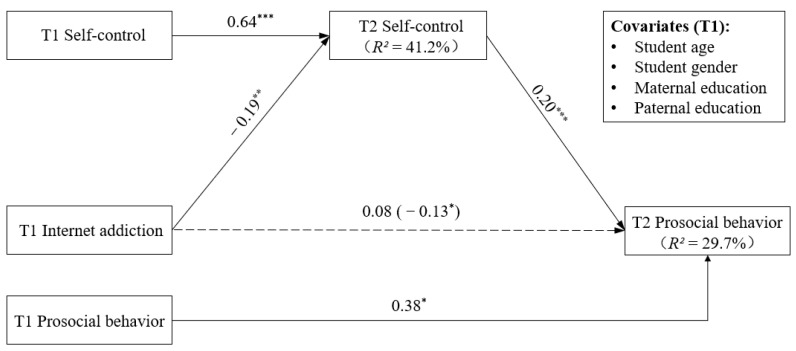
The mediating effect of self-control in the relationship between internet addiction and prosocial behavior. Note: The unstandardized coefficients are reported. * *p* < 0.05, *** *p* < 0.001. The dashed line indicates a non-significant coefficient. Same as below.

The mediation model (Table 2, Figure 2) fit the data well: *χ*^2^ = 18.23, *df* = 4, *p* < 0.001; RMSEA = 0.058 (90% CI = [0.033, 0.087]); CFI = 0.983; and SRMR = 0.015. After controlling for the covariates and T1 prosocial behavior, T1 internet addiction was significantly related to T2 self-control (*B* = −0.18, *SE* = 0.07, *p* =0.006) but was not significantly related to T2 prosocial behavior (*B* = 0.08, *SE* = 0.07, *p* = 0.270, *β* = 0.05). The results of the mediation analyses indicated that the mediating effect of T2 self-control was significant (*B* = −0.03, *SE* = 0.01, 95% CI = [−0.061, −0.011], *β* = −0.02). Thus, Hypothesis 2 was supported. 

### 3.5. Moderating Effects of Peer Rejection

**Figure 3:**
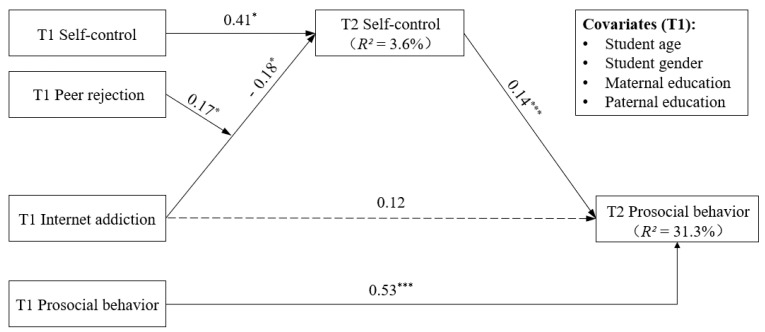
Moderated mediation model. * *p* < 0.05, *** *p* < 0.001.

**Figure 4:**
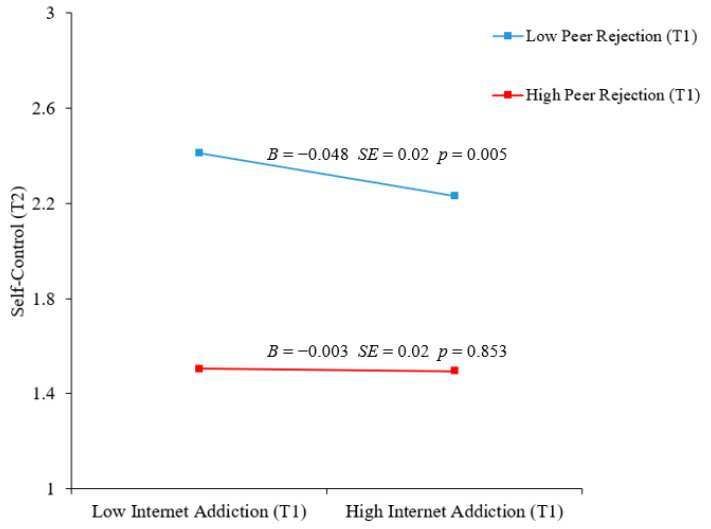
The relationship between internet addiction and self-control moderated by peer rejection.

The moderated mediation model fit the data well: *χ*^2^ = 28.31, *df* = 15, *p* < 0.05; RMSEA = 0.029 (90% CI = [0.011, 0.045]); CFI = 0.985; and SRMR = 0.017. The results are displayed in Table 3 and Figure 3, revealing that T1 peer rejection moderated the relationship between T1 internet addiction and T2 self-control (*B* = 0.17, *SE* = 0.79, *p* = 0.027, *β* = 0.07). As shown in Figure 4, the decomposition of the interaction using simple slope analyses revealed that, in contrast to individuals with high levels of peer rejection (*B* = −0.003, *SE* = 0.02, *p* = 0.853), for those with low levels of peer rejection, internet addiction more robustly negatively predicted self-control (*B* = −0.05, *SE* = 0.02, *p* = 0.005). It is only in the context of low peer rejection that internet addiction indirectly influences prosocial behavior through its impact on self-control. Importantly, the indirect effect of self-control was also moderated by peer rejection. As shown in Table 4, the results of the bias-corrected bootstrapped estimates indicated that the indirect effect of a low level of T1 internet addiction on T2 prosocial behavior via T2 self-control was significantly greater when T1 peer rejection was lower (*B* = −0.05, *SE* = 0.02, 95% CI = [−0.089, −0.020]) than when T1 peer rejection was higher (*B* = −0.003, *SE* = 0.02, 95% CI = [−0.036, 0.029]). In conclusion, these results support the idea that internet addiction has a greater negative association with adolescents’ prosocial behaviors via self-control when peer rejection is lower. Thus, Hypotheses 3a and 3b were supported.

## 4. Discussion

In contrast to previous cross-sectional investigations (Ma et al., 2011), this study filled gaps in the research by exploring the longitudinal association between internet addiction and prosocial behavior and the underlying mediating and moderating mechanisms among Chinese adolescents. Our results indicate that self-control mediates the association between internet addiction and adolescents’ prosocial behaviors, especially for adolescents with lower levels of peer rejection. The results provide valuable insights into the landscape of adolescents’ internet addiction in various ways, as elaborated upon below.

### 4.1. Association Between Internet Addiction and Prosocial Behavior

In this study, we employed a short-term longitudinal design while controlling for the baseline levels of prosocial behavior. The findings revealed a negative predictive relationship between internet addiction and adolescents’ prosocial behaviors six months later, confirming Hypothesis 1. This finding aligns with those of Collins and Freeman (2013), who similarly found that internet addiction negatively predicts prosocial behavior (Collins & Freeman, 2013; Ferguson, 2015). Internet addiction is posited to cause social skill deficits in real life (Chou et al., 2017) and social maladaptation between internet engagement and daily life (Zhao & Pan, 2022), which leads to tangible negative effects on adolescents’ psychological and social functioning (Su et al., 2020) and ultimately exerts a destructive effect on prosocial tendencies among adolescents (Yang et al., 2017).

### 4.2. The Mediating Role of Self-Control

Consistent with Hypothesis 2, the findings indicate that self-control mediates the connection between internet addiction and prosocial behavior. Our evidence suggests that internet addiction may influence adolescents’ self-control and consequently diminish their engagement in prosocial behaviors. This validation aligns with the results from previous cross-sectional research (Kim et al., 2017; Özdemir et al., 2014) and provides a further demonstration of self-control theory in longitudinal studies.

Internet addiction has evolved into a recognized social issue that impedes the normal development of adolescents (C. Li et al., 2014). Numerous studies have revealed that adolescents’ internet addiction has not only psychological and physical impacts (e.g., academic stress, depression, and poor sleep quality) (Jun & Choi, 2015; Stanković et al., 2021; Yen et al., 2007) but also negative effects on school burnout, academic stress, interpersonal interactions, etc. (Fumero et al., 2018; Jun & Choi, 2015; Salmela-Aro et al., 2017). This result aligns with previous findings related to the theory of self-control, in which self-control is adversely correlated with risk-taking behaviors (e.g., internet addiction) (Gottfredson & Hirschi, 1990). Internet addicts can easily become immersed in the internet, leading to impaired control abilities (Mehroof & Griffiths, 2010; Ng & Wiemer-Hastings, 2005). A study by Xiang et al. (2022) showed that enhancing self-control abilities among a sample of Chinese middle school students effectively deterred various issues, such as crime and internet addiction.

Additionally, the understanding of prosocial behavior may be affected by the limited resource model of self-control (Joosten et al., 2015). Most studies have consistently demonstrated an association between low self-control ability and increased negative behavior (e.g., risk-taking behaviors) (Liang et al., 2022; Tian et al., 2022). Nevertheless, this study revealed that a decrease in positive behavior (e.g., prosocial behavior) may be associated with low self-control. Empirical support for this perspective is limited but not entirely absent; for example, Barragan-Jason and Hopfensitz (2023) provided a demonstration of the negative connection between self-control and prosocial behavior. Considering the importance of self-control, Dou et al. (2019) demonstrated that individuals with high self-control exhibit more prosocial tendencies. This underscores the proposition that internet addiction significantly contributes to a decrease in adolescents’ self-control capacity, consequently reducing their prosocial behaviors. The results of this study emphasize the importance of enhancing adolescents’ self-control, addressing internet addiction concerns, and increasing their prosocial behaviors.

### 4.3. The Moderating Role of Peer Rejection

Consistent with Hypotheses 3a and 3b, the results show that self-control exerts a substantial negative effect on the internet addiction of adolescents, particularly among individuals with low levels of peer rejection. In the case of adolescents facing high peer rejection, internet addiction does not diminish their prosocial behaviors by reducing self-control. Conversely, for adolescents experiencing low peer rejection, a decrease in internet dependence correlates with elevated levels of self-control, leading to increased engagement in prosocial behavior.

These findings support an emerging line of research, as negative peer factors (characterized by high levels of peer rejection) attenuate the negative effect of internet addiction on self-control. A plausible explanation is that peer relationships exert greater influences on adolescents than family background (Loparo et al., 2023), and peer rejection has significant and enduring detrimental impacts on adolescent development (Huang et al., 2023; Loparo et al., 2023; Meldrum & Hay, 2012; Xiao & Qian, 2023). Social isolation or removal from peer relationships are typically the results of higher levels of peer rejection (Loparo et al., 2023), and adolescents who are disconnected from vital peer relationships may experience a loss of socialization skills with their peers (Gooren et al., 2011; Loparo et al., 2023). Moreover, rejection may lead to less self-control (Leary et al., 2006). Our findings support the notion that, regardless of the level of internet addiction, adolescents who experience high levels of peer rejection are likely to exhibit diminished self-control.

An explanation that could be considered is that individuals who experience peer rejection show greater sensitivity and susceptibility in adolescence (Stroud et al., 2009; Zhai et al., 2019). A low level of peer rejection seems to function as a protective factor, diminishing adolescents’ internet addiction, enhancing their self-control capabilities, and encouraging their prosocial behaviors. This explanation aligns with the theory of resilience. Fletcher (2013) considered protective factors (e.g., low levels of peer rejection) that protect individuals from adverse development outcomes in hazardous environments (e.g., internet addiction) and guide individuals towards a positive development process (e.g., prosocial behavior). However, the potential influence of other school and family factors in moderating internet addiction risk, as expected, should not be excluded. This prospect should be explored and re-examined in future studies.

In summary, the findings of this study contribute to the existing research by highlighting the importance of both environmental factors (i.e., peer rejection) and individual factors (i.e., self-control) in amplifying the negative effects of internet addiction. Furthermore, this study aligns with the individual-environment interaction model and suggests that individual and environmental factors frequently intertwine, influencing the physical and mental development of individuals rather than exerting effects alone in most cases (Rankin, 2019). Therefore, although peer rejection may directly impact adolescents’ internet addiction, the extent of this impact can vary on the basis of individuals’ levels of self-control. Adolescents invest more time in interacting with their peers in social settings than in other demographic settings, and the quality of peer relationships plays a pivotal role in fostering positive adaptive development among adolescents (King et al., 2018). The detrimental effects of inadequate peer relations on students’ internet addiction and self-control may persist. Thus, this study highlights the importance of acknowledging the power of peer influence and being vigilant regarding high levels of peer rejection, as it is a negative environmental factor influencing addictive behavior in adolescents.

### 4.4. Implications

This study aimed to provide a comprehensive framework for understanding the initiating elements and underlying mechanisms of adolescents’ prosocial behaviors and provide insight into solutions and strategies to support the healthy development of adolescents. First, the results show that the direct effect between internet addiction and prosocial behavior among Chinese adolescents was not significant after controlling for T1 prosocial behavior, which does not diminish the importance of internet addiction as a crucial risk factor. The mental health of internet addiction victims remains a pressing concern. Furthermore, internet addiction may decrease the propensity for prosocial behavior via self-control among Chinese adolescents. Thus, the findings of the current study underscore the need to strengthen adolescents’ self-control, decrease their internet addiction tendencies, and consequently enhance their prosocial behaviors. Second, the findings of this study highlight the potential benefits of focusing on adolescents with a low level of peer rejection, as this is associated with higher levels of self-control, which leads them to develop more prosocial behaviors. Finally, from the perspective of application, intervening in internet addiction to promote prosocial behavior is more effective for adolescents with a low level of peer rejection. Consequently, to foster prosocial behaviors among adolescents, it is imperative to effectively manage campus cyberbullying and intervene in internet addiction. Specifically, schools can implement measures against school bullying, such as establishing antibullying committees to reduce the occurrence of peer rejection. Additionally, parents and schools can collaborate to prevent adolescents from becoming addicted to the internet and increasing their self-control ability. Finally, both parents and schools can vigorously promote the benefits of prosocial behavior, thereby encouraging adolescents to actively engage in such behaviors.

### 4.5. Limitations

There are several limitations on this study. First, despite adopting a two-time longitudinal design and controlling for T1 internet addiction, importantly, the study remains correlational in nature, and firm causality cannot be conclusively inferred. Second, as the study applies specifically to junior and senior high school students in a particular region of China, generalizing the findings to Chinese adolescents as a whole should be performed cautiously. Third, the conclusions of the current study rely primarily on self-reports from adolescents, which may be influenced by shared method variance. To gain a thorough comprehension of the connection between internet addiction and prosocial behavior, future studies should employ different measurements (i.e., peer reports and parent reports). Fourth, this study primarily discussed the interaction between individuals and peers; thus, future research should explore the ways in which various environments (i.e., family, school) and individual factors synchronously influence adolescents’ self-control and prosocial behaviors.

## 5. Conclusions

This study demonstrated that internet addiction has a significant and unique impact on adolescents’ prosocial behaviors. After controlling for baseline prosocial behavior, self-control emerged as a key mediating pathway, shedding light on how internet addiction may reduce prosocial behavior. Importantly, the moderated mediation model highlighted that, among adolescents with low levels of peer rejection, reduced internet addiction was associated with enhanced self-control and increased engagement in prosocial behavior.

## Figures and Tables

**Table 1:** The means, standard deviations, and correlations of the study variables.

	*M*	*SD*	1	2	3	4	5	6
**Key variables**								
1. T1 Internet addiction	0.24	0.27						
2. T1 Peer rejection	2.54	0.90	0.11 ***					
3. T1 Self-control	3.14	0.57	−0.32 ***	−0.32 ***				
4. T2 Self-control	3.20	0.60	−0.29 ***	−0.24 ***	0.64 ***			
5. T1 Prosocial behavior	2.46	0.40	−0.25 ***	−0.16 ***	0.33 ***	0.24 ***		
6. T2 Prosocial behavior	2.44	0.45	−0.14 ***	−0.15 ***	0.24 ***	0.29 ***	0.52 ***	
**Covariates**								
Student age	14.80	1.61	0.03	0.11 **	−0.20 ***	−0.21 ***	−0.07 *	−0.07 *
Student gender	1.455	0.50	−0.31 ***	0.10 **	0.20	0.01	0.17 ***	0.16 ***
Father’s level of education	2.09	0.53	−0.02	−0.01	0.05	−0.01	0.03	0.02
Mother’s level of education	1.94	0.59	−0.05	−0.04	0.12 ***	0.03	0.07 *	0.02

Note: * *p* < 0.05, ** *p* < 0.01, *** *p* < 0.001. Student gender: 1 = males; 2 = females. Education: 1 = primary school; 2 = middle school; 3 = undergraduate; 4 = master or above. T1 = Time 1; T2 = Time 2. Same as below.

**Table 2:** Summary of direct and indirect effects.

Direct and Indirect Effects	Bias-Corrected Bootstrapped Estimates for Effects			
	*B*	*SE*	95% CI	*β*
**Direct Pathway**				
T1 IA → T2 PB	0.08	0.07	[−0.057, 0.231]	0.05
**Indirect Pathways**				
T1 IA → T2 SC → T2 PB	**−0.03**	**0.01**	**[−0.061, −0.011]**	**−0.02**

Note: The significant results are in bold. IA = internet addiction; PR = peer rejection; SC = self-control; PB = prosocial behavior. Same as below.

**Table 3:** Summary of direct and indirect effects.

	T2 Self-Control (*R*^2^ = 3.6%)				T2 Prosocial Behavior (*R*^2^ = 31.3%)			
	*B*	*SE*	*p*	*β*	*B*	*SE*	*p*	*β*
**Covariates**								
Student age					−0.001	0.01	0.926	−0.003
Student gender					**0.10**	**0.03**	**0.000**	**0.10**
Father’s level of education					0.01	0.03	0.726	0.01
Mother’s level of education					−0.01	0.03	0.644	−0.02
**Study variables**								
T1 Internet addiction	**−0.18**	**0.09**	**0.047**	**−0.08**	0.12	0.07	0.071	0.07
T1 Peer rejection	−0.45	0.35	0.187	−0.07				
T1 IA × T1 PR	**0.17**	**0.79**	**0.027**	**0.07**				
T2 Self-control					**0.14**	**0.03**	**0.000**	**0.19**
T1 Prosocial behavior					**0.53**	**0.03**	**0.000**	**0.48**

Note: Unstandardized regression coefficients are reported. Same as below.

**Table 4:** Conditional indirect effects of T1 internet addiction on T2 prosocial behavior via T2 self-control by levels of T1 peer rejection.

Levels of T1 Peer Rejection	*B*	*SE*	95% CI
**Low (M** − **1SD)**	**−0.05**	**0.02**	**[−0.089, −0.020]**
**Med (M)**	**−0.03**	**0.01**	**[−0.055, −0.003]**
High (M + 1SD)	−0.003	0.02	[−0.036, 0.029]
**Difference = High** − **Low**	**0.05**	**0.02**	**[0.007, 0.089]**

## Data Availability

The data sets used and/or analyzed during the current study are available from the corresponding author on reasonable request.

## Appendix A

**Table A1:** Measurement scales.

Questionnaire	Items
**Young Diagnostic Questionnaire (YDQ)** Keys: 0 = *No* 1 = *Yes*	Do you feel preoccupied with the Internet (think about previous online activity or anticipate next online session)?
	Do you feel the need to use the Internet with increasing amounts of time to achieve satisfaction?
	Have you repeatedly made unsuccessful efforts to control, cut back, or stop Internet use?
	Do you feel restless, moody, depressed, or irritable when attempting to cut down or stop Internet use?
	Do you stay online longer than originally intended?
	Have you jeopardized or risked the loss of a significant relationship, job, educational or career opportunity because of the Internet?
	Have you lied to family members, therapists, or others to conceal the extent of involvement with the Internet?
	Do you use the Internet as a way of escaping from problems or of relieving a dysphoric mood (e.g., feelings of helplessness, guilt, anxiety, depression)?
**Peer Rejection (PR)** Keys: 1 = *Totally disagree* 2 = *Disagree* 3 = *Not sure* 4 = *Agree* 5 = *Totally agree*	I sometimes feel as if some of my companions are ignoring me.
	I think some of my peers may not allow me to participate in their activities.
	I suspect some of my companions are trying to avoid me.
	I feel like some of my peers have left me out of the conversation.
	I think some of my companions are more likely to pretend that I don’t exist.
	I think some companions may not ask me to play together.
**Brief Self-Control Scale** **(BSCS)** Keys: 1 = *Not like me at all* 2 = *A little like me* 3 = *Not sure* 4 = *Like me* 5 = *Very much like me*	I am good at resisting temptation
	I have a hard time breaking bad habit.
	I am lazy.
	I say inappropriate things.
	I do certain things that are bad for me, if they are fun.
	I refuse things that are bad for me.
	I wish I had more self-discipline.
	People would say that I have iron self- discipline.
	Pleasure and fun sometimes keep me from getting work done.
	I do many things on the spur of the moment.
	I can work effectively toward long-term goals.
	Sometimes I can’t stop myself from doing something, even if I know it is wrong
	I often act without thinking through all the alternatives.
**Prosocial Behavior (PB)** Keys: 1 = *Not true* 2 = *Somewhat true* 3 = *Certainly true*	I try to be nice to other people, and I care about their feelings.
	I usually share with others (food, games, pens etc.).
	I am helpful if someone is hurt, upset or feeling ill.
	I am kind to younger children.
	I often volunteer to help others (parents, teachers, children).
